# Urethane Improves the Response of Auditory Neurons to Tone

**DOI:** 10.3389/fncel.2022.855968

**Published:** 2022-06-15

**Authors:** Bowan Huang, Linqing Yan, Yan Li, Wenhui Liu, Manhua Liu, Zhongju Xiao, Jinping Huang

**Affiliations:** ^1^Department of Physiology, School of Basic Medical Sciences, Southern Medical University, Guangzhou, China; ^2^Department of Anesthesiology, Shenzhen Traditional Chinese Medicine Hospital, The Fourth Clinical Medical College of Guangzhou University of Chinese Medicine, Shenzhen, China; ^3^The Seventh Affiliated Hospital, Southern Medical University, Foshan, China

**Keywords:** urethane, inferior colliculus, auditory sense, response to tone, improvement

## Abstract

Urethane has little effect on nervous system and is often used in neuroscience studies. However, the effect of urethane in neurons is not thoroughly clear. In this study, we investigated changes in neuron responses to tones in inferior colliculus during urethane anesthesia. As urethane was metabolized, the best and characteristic frequencies did not obviously change, but the minimal threshold (MT) remained relatively stable or was elevated. The frequency tuning bandwidth at 60 dB SPL (BW_60dBSPL_) remained unchanged or decreased, and the average evoked spike of effective frequencies at 60 dB SPL (ES_60dBSPL_) gradually decreased. Although the average evoked spike of effective frequencies at a tone intensity of 20 dB SPL above MT (ES_20dBSPLaboveMT_) decreased, the frequency tuning bandwidth at a tone intensity of 20 dB SPL above MT (BW_20dBSPLaboveMT_) did not change. In addition, the changes in MT, ES_60dBSPL_, BW_60dBSPL_, and ES_20dBSPLaboveMT_ increased with the MT in pre-anesthesia awake state (MT_pre−anesthesiaawake_). In some neurons, the MT was lower, BW_60dBSPL_ was broader, and ES_60dBSPL_ and ES_20dBSPLaboveMT_ were higher in urethane anesthesia state than in pre-anesthesia awake state. During anesthesia, the inhibitory effect of urethane reduced the ES_20dBSPLaboveMT_, but did not change the MT, characteristic frequency, or BW_20dBSPLaboveMT_. In the recording session with the strongest neuron response, the first spike latency did not decrease, and the spontaneous spike did not increase. Therefore, we conclude that urethane can reduce/not change the MT, increase the evoked spike, or broaden/not change the frequency tuning range, and eventually improve the response of auditory neurons to tone with or without “pushing down” the tonal receptive field in thresholding model. The improved effect increases with the MT_pre−anesthesiaawake_ of neurons. The changes induced by the inhibitory and improved effects of urethane abide by similar regularities, but the change directions are contrary. The improvement mechanism may be likely due to the increase in the ratio of excitatory/inhibitory postsynaptic inputs to neurons.

## Introduction

Urethane is a common general anesthetic in neuroscience studies (e.g., auditory studies) (Maggi and Meli, 1986; Yang et al., 2021). Hearing is the final lost sense during anesthesia (Ghoneim and Block, 1992). Subcortical structure may play an important role in restlessness during anesthesia recovery (Sachdev and Kruk, 1996). However, the effect of urethane on neurons of the auditory subcortical structure is not thoroughly clear. Solving this problem will help to interpret the research data under urethane anesthesia and to understand the mechanism of general anesthesia.

General anesthetics can increase the amplitude of auditory evoked potentials, which is due to the increased synchronization of electrical activities of neurons (Church and Shucard, 1987; Huang et al., 2015). In addition, there is an excitatory phenomenon during anesthesia induction (Guedel, 1937) and recovery (Xu et al., 2017). It has been speculated that the excitatory phenomenon during anesthesia recovery may be attributed to an increase in the excitability of subcortical neurons (Sachdev and Kruk, 1996). In brief, general anesthetics may improve the response of neurons.

In previous studies, general anesthetics usually inhibited the response of auditory neurons (Syka et al., 2005; Felix et al., 2012), which was reflected by decreasing the evoked spike (ES) (Albrecht and Davidowa, 1989; Capsius and Leppelsack, 1996), elevating the response threshold of neurons to sound (van Looij et al., 2004), and narrowing the frequency tuning range (Gaese and Ostwald, 2001). Urethane is usually assumed to have little direct inhibitory effect on physiological functions (Maggi and Meli, 1986). Therefore, based on the above analysis, we hypothesize that, contrary to the inhibitory effect, urethane can increase the ES, reduce the minimal threshold (MT) or broaden the frequency tuning range, and eventually improve the response of auditory neurons.

To test our hypothesis, under urethane anesthesia, we applied tone as an acoustic stimulus with a delivery rate of 2 Hz and recorded the spike of neurons in the central nucleus of the inferior colliculus (ICC). In this manner, we attempted to further investigate the effect of urethane on neurons in the ICC.

## Materials and Methods

### General

All experimental procedures used in this study were approved by the Animal Care and Use Committee of Southern Medical University (No. 2014-037, Chairperson Prof. Weiwang Gu). Twenty female BALB/c mice (aged 4–6 weeks) with normal hearing were purchased from the Experimental Animal Center of Southern Medical University, Guangzhou, China. All mice were used according to the guidelines set by the Animal Care and Use Committee of Southern Medical University. The methods for surgical procedures, acoustic stimulation, data acquisition and processing in this study were similar to those in our previous studies (Huang et al., 2015, 2019).

### Surgical Preparation

Pentobarbital (60–70 mg/kg, i.p.) was used to anesthetize each mouse. Under sterile conditions, we exposed the skull of each mouse and inserted a reference electrode under the prefrontal bone. To immobilize the head, we glued a 1.5-cm-long nail on the mouse's skull with dental cement. Then, to record the spike of neurons, we opened a 2 × 2-mm^2^ bone window on the inferior colliculus (IC) (anteroposterior = −5.02 mm and mediolateral = 1.13 mm from bregma) to expose the brain tissue *via* a minielectric drill under a surgical microscope (WPI, USA). Next, vaseline plastic wrap and tissue were applied to cover the exposed brain, and the mouse was put back into its cage for recovery. During the recovery process, the mouse was free to acquire food and water.

### Acoustic Stimulation

A Tucker-Davis Technologies System 3 (TDT 3, Tucker-Davis Technologies, Alachua, FL, USA) was applied to generate and deliver the acoustic stimuli (pure tone and noise bursts), which were synthesized by a real-time processor (RP 2.1) and a custom-made program (written with RPvdsEx software) and put in a programmable attenuator (PA5) to adjust the intensity. The synthesized signals were amplified by an electrostatic speaker driver (ED1) and presented to the mouse *via* a calibrated closed acoustic delivery system comprising two TDT EC1 speakers with couplers. The sound parameters were regulated by Brain Ware software.

### Data Acquisition

After at least 2 days of recovery, the prepared mouse was transferred to the anti-vibration table again, and its head was fixed. The covers and dura on the exposed IC were cleared. To record acoustic evoked spikes, a foursquare tungsten four-microelectrode array (impedance: 2–4 MΩ) was used. To search suitable recording sites (there were at least two recording sites, whose neuron responses had a signal-noise ratio >4:1), with a microdriver (Narishige MO-10, Japan), the microelectrode array was slowly inserted perpendicularly into the ICC while simultaneously presenting 50-ms noise bursts (60 dB SPL). The spikes were amplified 10,000 times, filtered by a bandpass of 300–3,000 Hz with a digital amplifier RA16 and recorded and displayed with Brain Ware software.

Once suitable recording sites were found, to identify the characteristic frequency (CF) of each recording site, the first frequency-intensity scan (F-I scan) was carried out, in which pure tone bursts [2–64 kHz at 0.1 octave intervals (Acoustic frequency = 2,000 × 2^i^, *i* = 0: 0.1: 5), 20–90 dB SPL in 10 dB SPL steps, 50 ms duration, 5 ms rise-fall time] were randomly presented at a rate of 2/s with 3 repetitions. Then, the mouse was anesthetized with urethane (1,250 mg/kg i.p.).

Five minutes later, the second and third F-I scans were carried out and repeated every 10 min until the mouse awoke (i.e., the mouse limbs autonomously moved). In the second F-I scan, pure tone bursts (four CFs of recording sites, 60 dB SPL, 50 ms duration, 5 ms rise-fall time) were randomly presented at a rate of 2/s with 10 repetitions. In the third F-I scan, pure tone bursts (2–32 kHz, 3–48 kHz, or 4–64 kHz at 0.1 octave intervals, 20–90 dB SPL in 10 dB SPL steps, 50 ms duration, 5 ms rise-fall time) were randomly presented at a rate of 2/s with 3 repetitions. In addition, as a control, four mice were not anesthetized with urethane and were recorded for 300 min in the same way. During the second and third F-I scans, the spikes (time window: 500 ms after stimulus onset) and the corresponding acoustic stimuli parameters in each recording session were recorded and saved in a DAM file.

During recording, the exposed IC was treated with physiological saline continuously to prevent the tissue from drying, and the pinnae were maintained as in normal awake mice. The rectal temperature was monitored and was not allowed to vary by more than 0.4°C *via* a homemade homeothermic blanket (Jones et al., 1980; Rossi and Britt, 1984). After the recording, the tungsten microelectrodes were pulled out from the ICC and dyed with pontamine sky blue. Then, the tungsten microelectrodes were inserted into the ICC at the same depth. After that, the brain tissue was cut into slices to confirm the recording locations in the ICC according to the atlas for the mouse brain (anteroposterior = −4.96 to −5.22 mm and mediolateral = 0.5–1.5 mm from bregma, depth = 0.25–1.8 mm). Data obtained outside the ICC were abandoned.

### Data Processing

For each recording channel, *via* Offline Sorter (Plexon), semiautomatic spike sorting was carried out. *Via* a T-Dist E-M scan algorithm (scan over a range of 10–30 degree of freedom), semiautomated clustering was performed according to the first three principal components of the spike waveform or Peak-Valley values, then evaluated with sort quality metrics. Clusters with isolation distance <20 and L-Ratio >0.1 were discarded. Spike clusters were classified as single units only if the waveform signal-noise ratio exceeded 4 and the inter-spike intervals exceeded 1.2 ms for >99.5% of the spikes (Shen et al., 2022).

**Figure 1:**
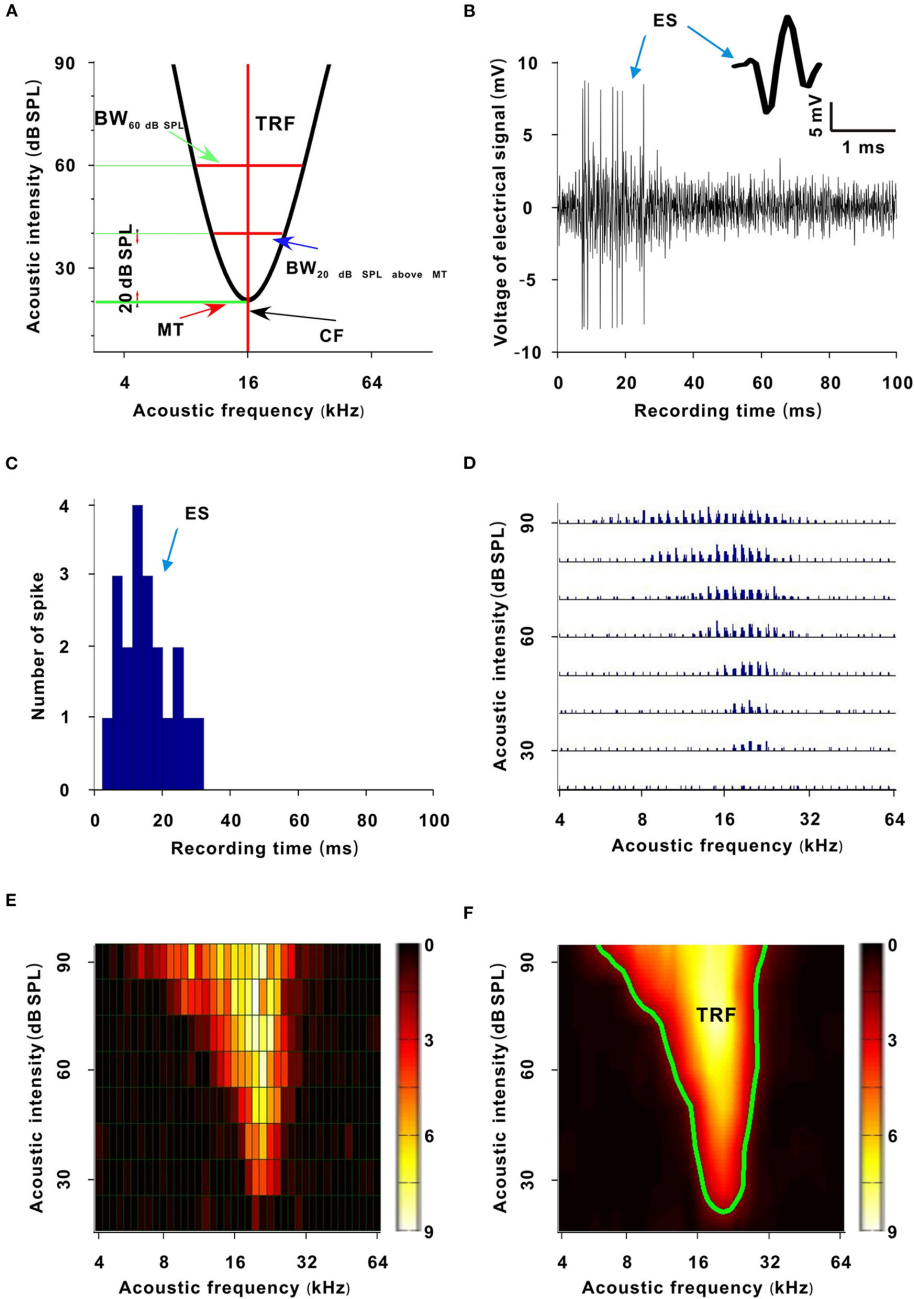
Sketch map and generative process of the TRF. **(A)** Sketch map of the TRF. The abscissa is acoustic frequency and the ordinate is acoustic intensity (the same meaning in **(D–F)** and Figures 2, 4, 6). The area enveloped by the black curve is the TRF. The acoustic intensity corresponding to the bottom green transverse line is the MT. The acoustic frequency corresponding to the red vertical line is the CF. The width of the upper red transverse line is the BW_60 dB SPL_. The width of the lower red transverse line is the BW_20 dB SPL above MT_. **(B)** Recorded electrical signals after a tone stimulus (100 ms). The inset shows a spike shape. **(C)** PSTH (three repetitive tones, 3 ms/bar). **(D)** TRF. Each small trace represents a PSTH of a tone stimulus. **(E)** TRF displayed with a pseudocolor map. Each small grid represents a tone stimulus. The color of a small grid represents the averaged ES number of the corresponding tone. **(F)** Smoothed TRF from **(E)**. The color of each coordinate point represents the averaged ES number of the corresponding tone (the same meaning in Figures 2, 4, 6). The green curve is the contour of the TRF.

Within 50 ms before tone onset, the spikes of a neuron were used to calculate the spontaneous spike (SS) rate. After tone onset, when the spike rate of a neuron was more than two times greater than the standard deviation of the SS rate (Liang et al., 2014), these spikes were regarded as tone ESs (Figures 1B,C). For each recording session, the ES corresponding to tones in the second F-I scan was used to obtain the first-spike latency (FSL) (Tan et al., 2008). The FSL was defined as the time from tone onset to the occurrence time of the first spike.

**Figure 2:**
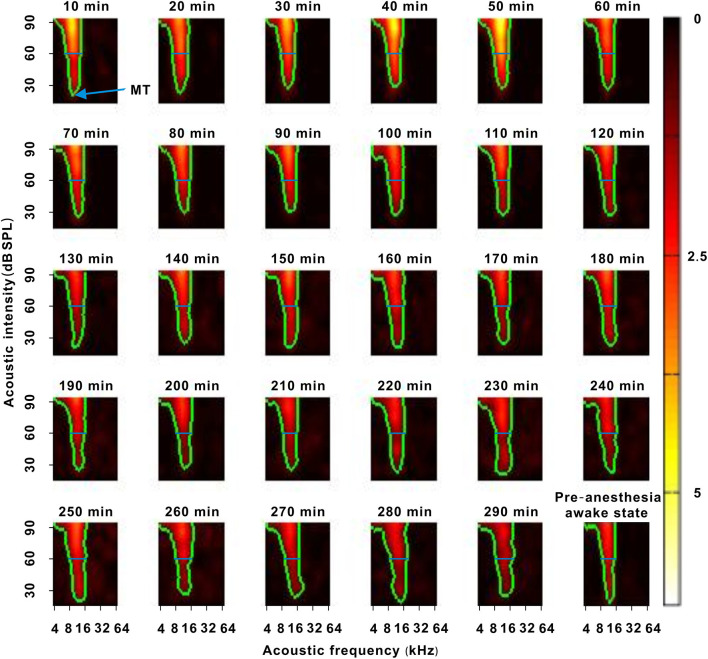
A representative neuron with a change in response intensity. The 10–290 min was the time after urethane anesthesia. Each green curve was the contour of the TRF. The blue arrow labeled the location of the MT. The blue lines labeled the location of 60 dB SPL. The last pseudocolor map in the bottom right corner was obtained under the pre-anesthesia awake state.

**Figure 3:**
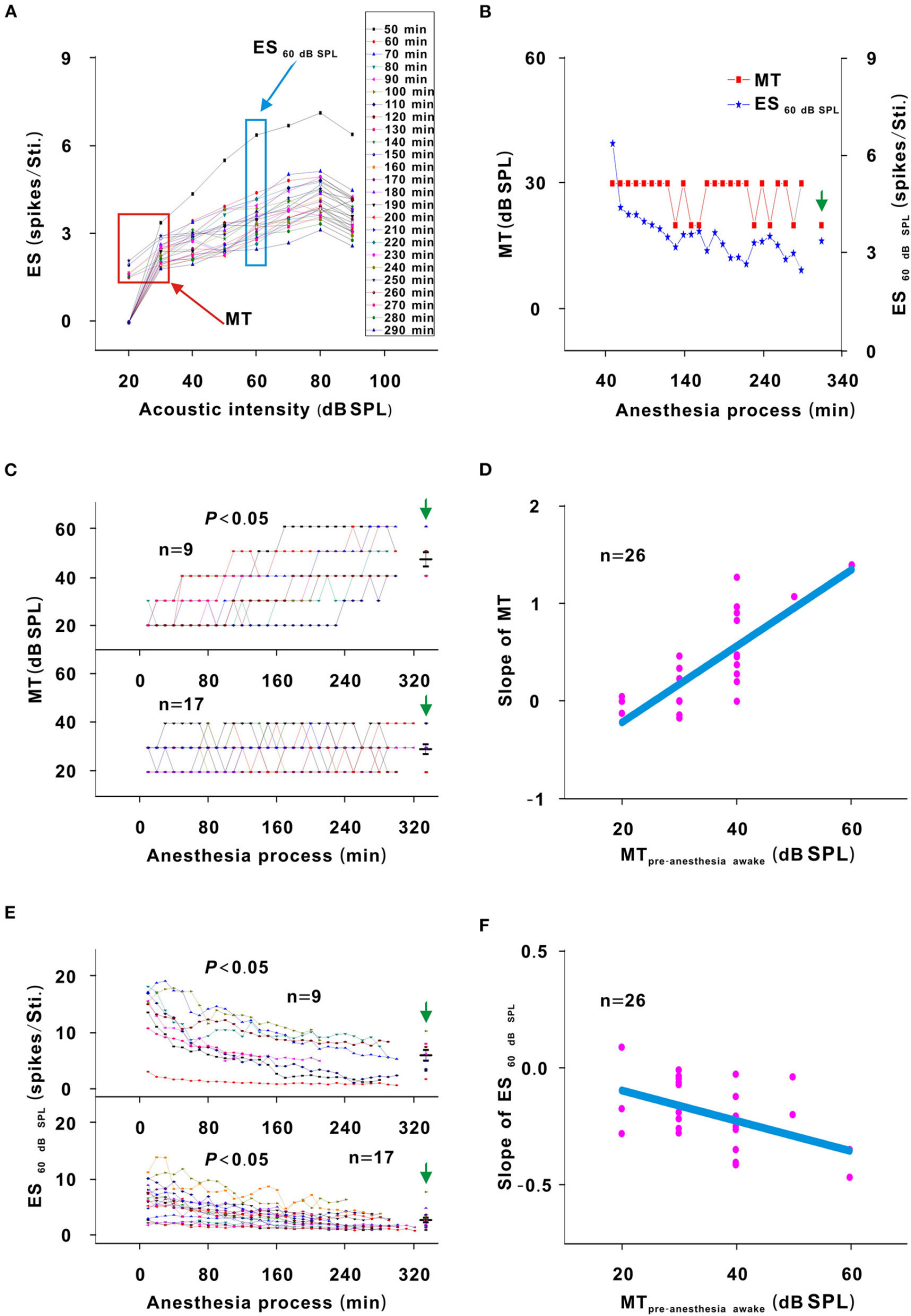
Changes in the response intensity of neurons. **(A)** ES changes with acoustic intensity. The red and blue frames indicated the MT and ES_60 dB SPL_ of all recording sessions, respectively. **(B)** MT and ES_60 dB SPL_ changes with anesthesia time (red curve and left ordinate for the MT; blue curve and right ordinate for the ES_60 dB SPL_), hereafter, the MT- and ES_60 dB SPL_-time curves. The vertical green arrow labeled the data recorded under the pre-anesthesia awake state (the same meaning in all following figures). **(C)** Summary of the MT-time curves in neurons_changed MT_ (upper curves) and neurons_unchangedMT_ (lower curves) [Upper curves: first four recording sessions vs. middle four recording sessions vs. last four recording sessions vs. pre-anesthesia awake state (21.944 ± 3.254) vs. (31.944 ± 9.825) vs. (45.278 ± 10.712) vs. (46.667 ± 8.660) dB SPL, *F* = 16.695, *P* = 1.046 × 10^−6^, one-way ANOVA, Multiple comparisons (Tamhane's T2): *P* = 2.271 × 10^−6^ for first four recording sessions vs. last four recording sessions, *P* = 8.428 × 10^−7^ for first four recording sessions vs. pre-anesthesia awake state; Lower curves: first four recording sessions vs. middle four recording sessions vs. last four recording sessions vs. pre-anesthesia awake state (26.471 ± 5.234) vs. (27.353 ± 5.760) vs. (28.824 ± 5.668) vs. (29.412 ± 8.269) dB SPL, *F* = 0.764, *P* = 0.518, one-way ANOVA]. The first recording session was the recording session with the strongest neuron response (highest ES) (e.g., the strongest recording session was at 50 min in Figure 2). The same meaning applied in **(E)** and Figures 5C,D, 7D–F, 8A,B. The middle black transverse line represented the mean, and the upper/lower black transverse lines represented the mean ± 1 SD (the same meaning applied in all the following figures). **(D)** Scatterplot for slopes of the MT-time curves against the MT_pre−anesthesia awake_ and the fitting curve to a linear equation (*P* = 2.112 × 10^−7^, *R*^2^ = 0.644, intercept = −1.000, slope = 0.039). **(E)** ES_60 dB SPL_-time curves in neurons_changed MT_ (upper curves) and neurons_unchanged MT_ (lower curves) [Upper curves: first four recording sessions vs. middle four recording sessions vs. last four recording sessions vs. pre-anesthesia awake state (12.457 ± 4.756) vs. (7.092 ± 3.672) vs. (5.171 ± 3.154) vs. (5.801 ± 2.718) spikes/stimulus (Sti.), *F* = 7.406, *P* = 0.001, one-way ANOVA, Multiple comparisons (LSD): *P* = 1.835 × 10^−4^ for first four recording sessions vs. last four recording sessions, *P* = 5.143 × 10^−4^ for first four recording sessions vs. pre-anesthesia awake state; Lower curves: first four recording sessions vs. middle four recording sessions vs. last four recording sessions vs. pre-anesthesia awake state (5.772 ± 2.964) vs. (3.532 ± 1.801) vs. (2.487 ± 1.459) vs. (2.649 ± 1.617) spikes/Sti., *F* = 9.275, *P* = 3.533 × 10^−5^, one-way ANOVA, Multiple comparisons (Tamhane's T2): *P* = 0.003 for first four recording sessions vs. last four recording sessions, *P* = 0.005 for first four recording sessions vs. pre-anesthesia awake state]. **(F)** Scatterplot for slopes of ES_60 dB SPL_-time curves against the MT_pre−anesthesia awake_ and the fitting curve to a linear equation (*P* = 0.009, *R*^2^ = 0.181, intercept = 0.051, slope = −0.013).

The ESs corresponding to tones in the third F-I scan were counted. These ESs for each tone were applied to plot the frequency-intensity tonal receptive field (TRF), which could be displayed with a poststimulus spike time histogram (PSTH) or pseudocolor map *via* a custom-made MATLAB program (Figures 1D–F) (Sun et al., 2010). A cubic spline interpolation algorithm was used to smooth the TRF (Figure 1F). The boundary (envelope) of the TRF (i.e., frequency-intensity tuning curve) was obtained based on the continuity of tone-evoked responses along the frequency and intensity domains *via* another custom-made MATLAB program and was defined at the level of 30% of the maximum spike response (Figure 1A, black curve and Figure 1F, green curve) (Liang et al., 2014). The tone intensity at the tip of the frequency-intensity tuning curve was set as the MT of the TRF (Figure 1A) (Liang et al., 2014). The acoustic frequency or the logarithmic center of the frequency range at the MT was defined as the CF of the recorded neuron (Figure 1A) (Liang et al., 2014). For any tone with a fixed acoustic intensity, if its acoustic frequency was located in the TRF, this frequency was an effective frequency. We defined the range of effective frequencies (i.e., frequency tuning bandwidth) at 60 dB SPL as BW_60dBSPL_ (Figure 1A), and we defined the frequency tuning bandwidth at a tone intensity of 20 dB SPL above MT as BW_20dBSPLaboveMT_ (Figure 1A). The average ES of effective frequencies at 60 dB SPL was defined as the ES_60dBSPL_. The average ES of effective frequencies at a tone intensity of 20 dB SPL above MT was defined as ES_20dBSPLaboveMT_ (Liang et al., 2014). The acoustic frequency with the largest ES_60 dB SPL_ was defined as the best frequency (BF) (Huang et al., 2015). The BW_60 dB SPL_ and BW_20 dB SPL above MT_ were presented with octaves (e.g., the difference between two adjacent acoustic frequencies was 0.1 octave). After obtaining these variables, we plotted them against anesthesia time. For all neurons presented in those figures (i.e., **Figures 3C,E**, **5C,D**, **7D–F**, **8A,B**), the first recording session of each curve was the recording session with the strongest neuron response (highest ES) (e.g., the strongest recording session was at 50 min in Figure 2). In **Figure 9**, the last recording session of each curve was the recording session with the strongest neuron response.

### Statistical Analysis

All statistical analyses were performed in SPSS statistical software (version 13). The measurement data were presented as the mean ± SD. To analyze the change in each variable with recording time, the average values of first four recording sessions, middle four recording sessions and last four recording sessions after urethane anesthesia, and the average values of pre-anesthesia awake state were calculated and tested with one-way ANOVA. For one-way ANOVA, LSD or Tamhane's *T*2-test was used for multiple comparisons. For two-group comparisons (e.g., first recording session vs. recording session with strongest response in **Figure 9**), paired or unpaired *t-*tests were applied to test significance. A *P-*value < 0.05 was deemed significant.

## Results

We recorded electrophysiological data in 31 neurons of 20 mice located 250–1,800 μm below the surface of the ICC. The recording time was 210–360 min. The MT under the pre-anesthesia awake state (MT_pre−anesthesiaawake_) of neurons was 20–60 (33.548 ± 11.416) dB SPL. The CF of neurons was 8–34.296 (19.646 ± 9.760) kHz.

### Effect of Urethane on the Response Intensity of Neurons

In each neuron recording session, the spike TRF was mapped. In a representative neuron, the area of the TRF remained stable during anesthesia (Figure 2). According to the color change of the TRF, the response of this neuron first increased from 10 to 50 min and then gradually decreased from 50 to 290 min after anesthesia (Figure 2). From 50 to 290 min, the MT basically remained unchanged, and the ES_60dBSPL_ gradually decreased (Figures 3A,B). However, as urethane was metabolized, the MT gradually increased in 9 neurons (*P* < 0.05, difference between MT at 10 min after anesthesia and MT of last recording session were equal to or >20 dB SPL), and did not obviously change in 17 neurons (*P* > 0.05) (Figure 3C, difference between MT at 10 min after anesthesia and MT of last recording session were <20 dB SPL).

For neurons with changed MT values (neurons_changedMT_), the MT_pre−anesthesiaawake_ were 40–60 dB SPL, which were higher than those (20–40 dB SPL) of neurons without obvious MT change (neurons_unchangedMT_) (Figure 3C, unpaired *t*-test, *t* = −5.700, *P* = 7.153 × 10^−6^). For neurons_changedMT_ (Figure 3E, upper curves), the ES_60 dB SPL_ decreases (*P* < 0.05) seemed to be more obvious than those (*P* < 0.05) in neurons_unchanged MT_ (Figure 3E, lower curves). When the ES_60 dB SPL_-or MT-time curves were fit with a linear equation, the slope of each curve was obtained. As the MT_pre−anesthesia awake_ increased, the absolute value of the slopes gradually increased (Figures 3D,F).

In addition, the MT of neurons_unchanged MT_ did not obviously change (*P* > 0.05), and the MT of neurons_changed MT_ was lower (*P* < 0.05) and the ES_60 dB SPL_ of all neurons was higher (*P* < 0.05) in the first four recording sessions than in the pre-anesthesia awake state (Figures 3C,E). Therefore, urethane could excite neurons, and the excitatory effect was more obvious in neurons with high MT_pre−anesthesia awake_ values.

### Effect of Urethane on the Frequency Tuning of Neurons

**Figure 4:**
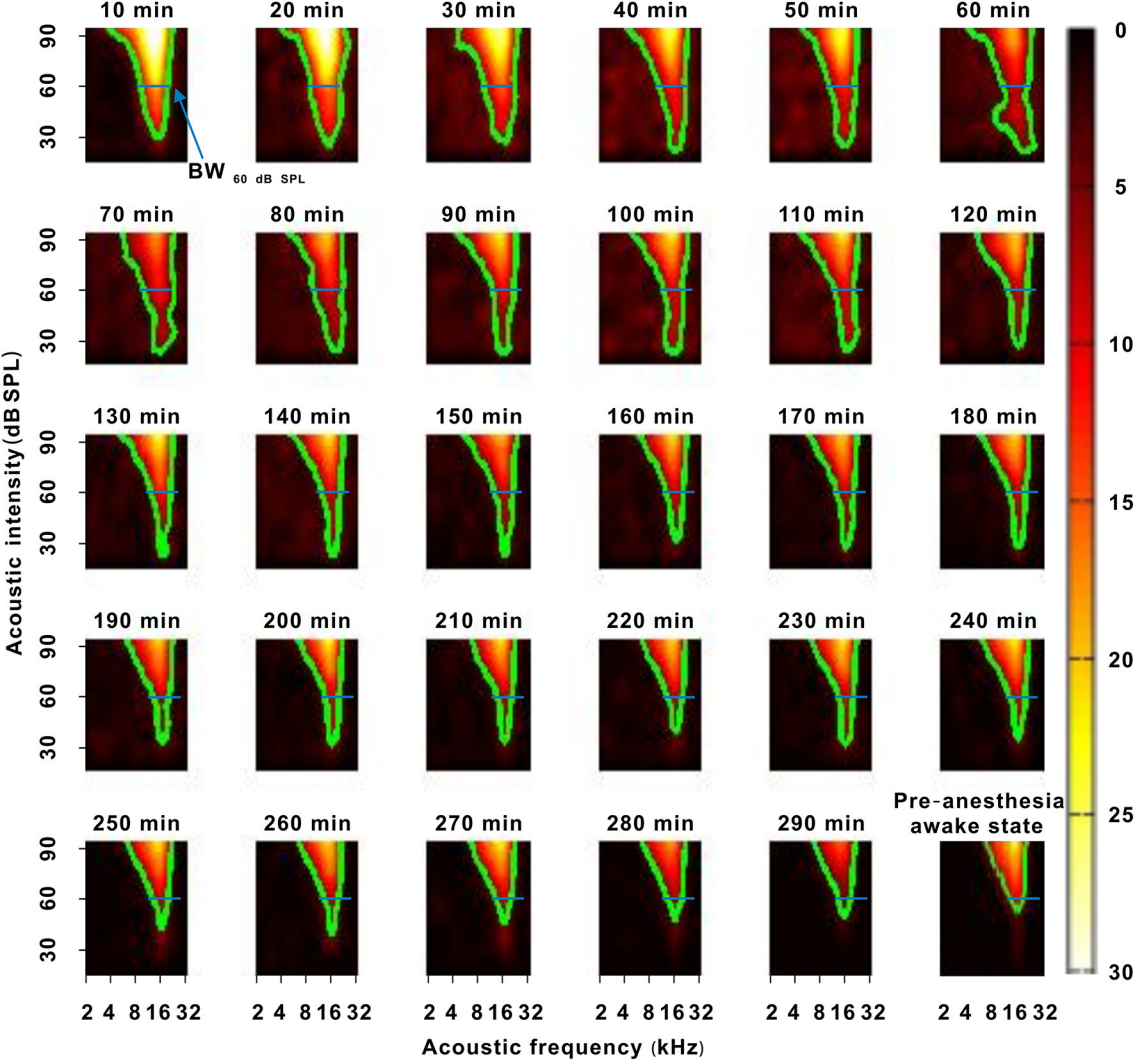
A representative neuron with a change in frequency tuning. The 10–290 min was the time after urethane anesthesia. Each green curve was the contour of the TRF. The width of the blue transverse line represented the BW_60 dB SPL_ in the first recording session. The last pseudocolor map in the bottom right corner was obtained under the pre-anesthesia awake state.

**Figure 5:**
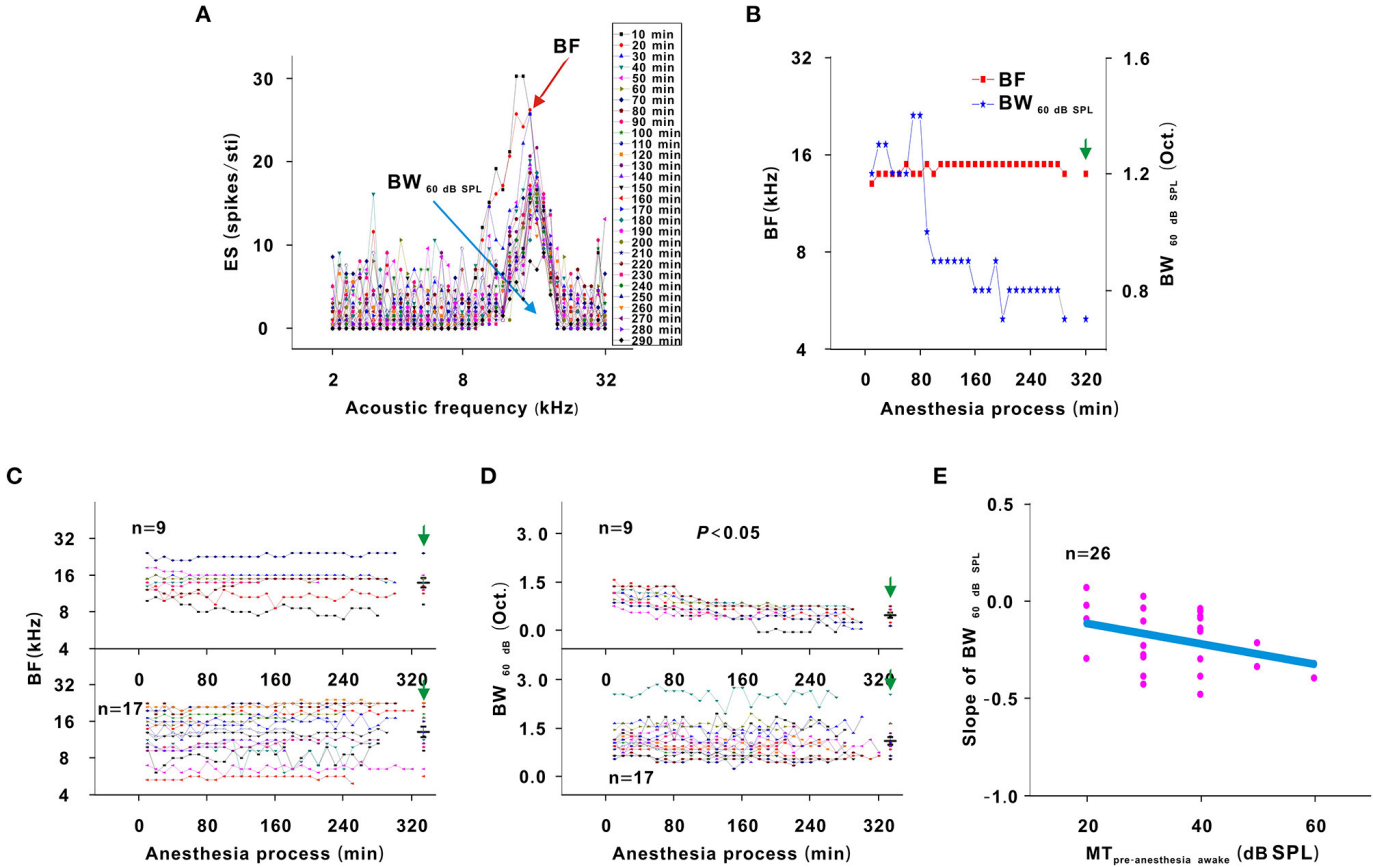
Changes in the frequency tuning of neurons. **(A)** ES changes with acoustic frequency. The highest dot of each curve labeled BF (red arrow). The blank width of each curve was the BW_60 dB SPL_ (blue arrow). **(B)** BF and BW_60 dBSPL_ changes with anesthesia time (red curve and left ordinate for the BF, blue curve and right ordinate for the BW_60 dB SPL_), hereafter, the BF- and BW_60dBSPL_-time curves. **(C)** Summary of the BF-time curves in neurons_changed MT_ (upper curves) and neurons_unchanged MT_ (lower curves) [Upper curves: first four recording sessions vs. middle four recording sessions vs. last four recording sessions vs. pre-anesthesia awake state (14.552 ± 3.697) vs. (14.540 ± 4.136) vs. (14.557 ± 4.290) vs. (14.406 ± 4.246) kHz, *F* = 0.003, *P* = 1.000, one-way ANOVA; Lower curves: first four recording sessions vs. middle four recording sessions vs. last four recording sessions vs. pre-anesthesia awake state (13.033 ± 4.904) vs. (14.126 ± 5.248) vs. (14.407 ± 5.569) vs. (14.105 ± 5.291) kHz, *F* = 0.262, *P* = 0.878, one-way ANOVA]. **(D)** Summary of the BW_60 dB SPL_-time curves in neurons_changed MT_ (upper curves) and neurons_unchanged MT_ (lower curves) [Upper curves: first four recording sessions vs. middle four recording sessions vs. last four recording sessions vs. pre-anesthesia awake state (1.075 ± 0.231) vs. (0.678 ± 0.175) vs. (0.511 ± 0.217) vs. (0.522 ± 0.233) octave (Oct.), *F* = 13.492, *P* = 7.363 × 10^−6^, one-way ANOVA, Multiple comparisons (LSD): *P* = 3.922 × 10^−6^ for first four recording sessions vs. last four recording sessions, *P* = 5.395 × 10^−6^ for first four recording sessions vs. pre-anesthesia awake state; Lower curves: first four recording sessions vs. middle four recording sessions vs. last four recording sessions vs. pre-anesthesia awake state (1.193 ± 0.455) vs. (1.102 ± 0.489) vs. (1.091 ± 0.504) vs. (1.171 ± 0.484) Oct, *F* = 0.183, *P* = 0.907, one-way ANOVA]. **(E)** Scatterplot for slopes of BW_60dBSPL_-time curves against MT_pre−anesthesia awake_ and the fitting curve to a linear equation (*P* = 0.001, *R*^2^ = 0.104, intercept = −0.007, slope = −0.005).

In another representative neuron, as urethane was metabolized, the area of the TRF gradually decreased. As seen from the color change in the TRF, the response of this neuron gradually decreased from 10 to 290 min after anesthesia (Figure 4). Although there were some slight variations in the initial several recording sessions, the BF did not change in the latter recording sessions. The BW_60 dB SPL_ showed a gradual decreasing trend with anesthesia time (Figure 5A). This is more clearly shown in the BF- and BW_60 dB SPL_-time curves (Figure 5B).

For all neurons, whether neurons had changed or unchanged MTs, the BF remained relatively stable during anesthesia (*P* > 0.05; Figure 5C). However, the BW_60 dB SPL_ did not obviously change in neurons_unchanged MT_ (*P* > 0.05) and gradually narrowed in neurons_changed MT_ (*P* < 0.05; Figure 5D). Similarly, a linear equation was used to fit the BW_60 dB SPL_-time curve to obtain the slope, which decreased with the MT_pre−anesthesia awake_ (Figure 5E). In first four recording sessions, the BF of all neurons and BW_60 dB SPL_ of neurons_unchanged MT_ were similar to those observed in the pre-anesthesia awake state (*P* > 0.05), and the BW_60 dB SPL_ of neurons_changed MT_ was broader than it was in the pre-anesthesia awake state (*P* < 0.05; Figures 5C,D). That is, urethane can broaden the frequency tuning of neurons. The BW_60 dB SPL_ was more easily changed by urethane in neurons with high MT_pre−anesthesia awake_ values.

### Effect of Urethane on the TRF of Neurons

The TRF is a fundamental functional property of auditory neurons (Sun et al., 2013). There are three different models (thresholding, summation/subtraction, and multiplication/division models) to describe the change in the TRF (Xiong et al., 2013). For thresholding model, the contour of the TRF was pushed down/up without change in the shape of the bottom. For multiplication/division model, the contour of the TRF was pushed down/up *via* multiplying/dividing an effect. For summation/subtraction model, the contour of the TRF was pushed down/up *via* summating/subtracting an effect (Figure 7A).

**Figure 6:**
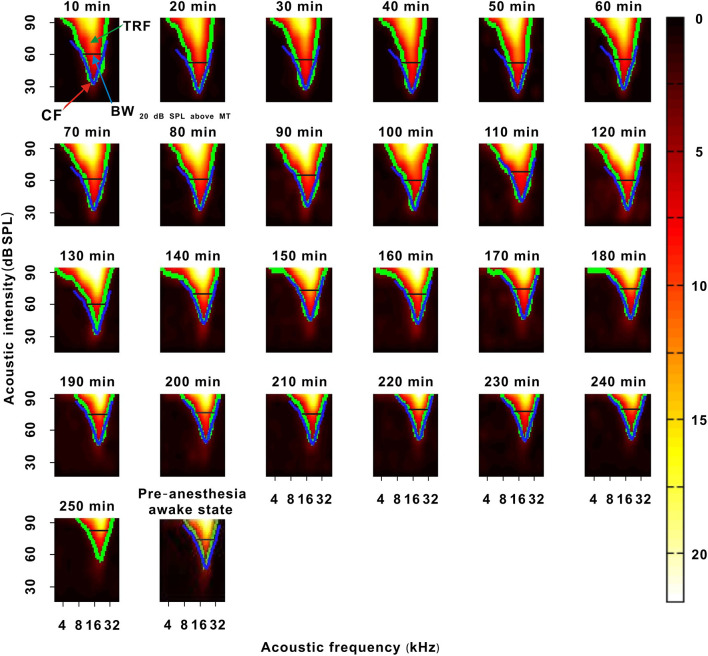
A representative neuron with a change in the TRF. The 10–250 min was the time after urethane anesthesia. Each green curve was the contour of the TRF. The blue curve showed the contour of the TRF in the last recording session. The width of the black transverse line was BW_20 dB SPL above MT_ in the last recording session. The red arrow labeled the location of CF. The last pseudocolor map on the bottom was obtained under the pre-anesthesia awake state.

**Figure 7:**
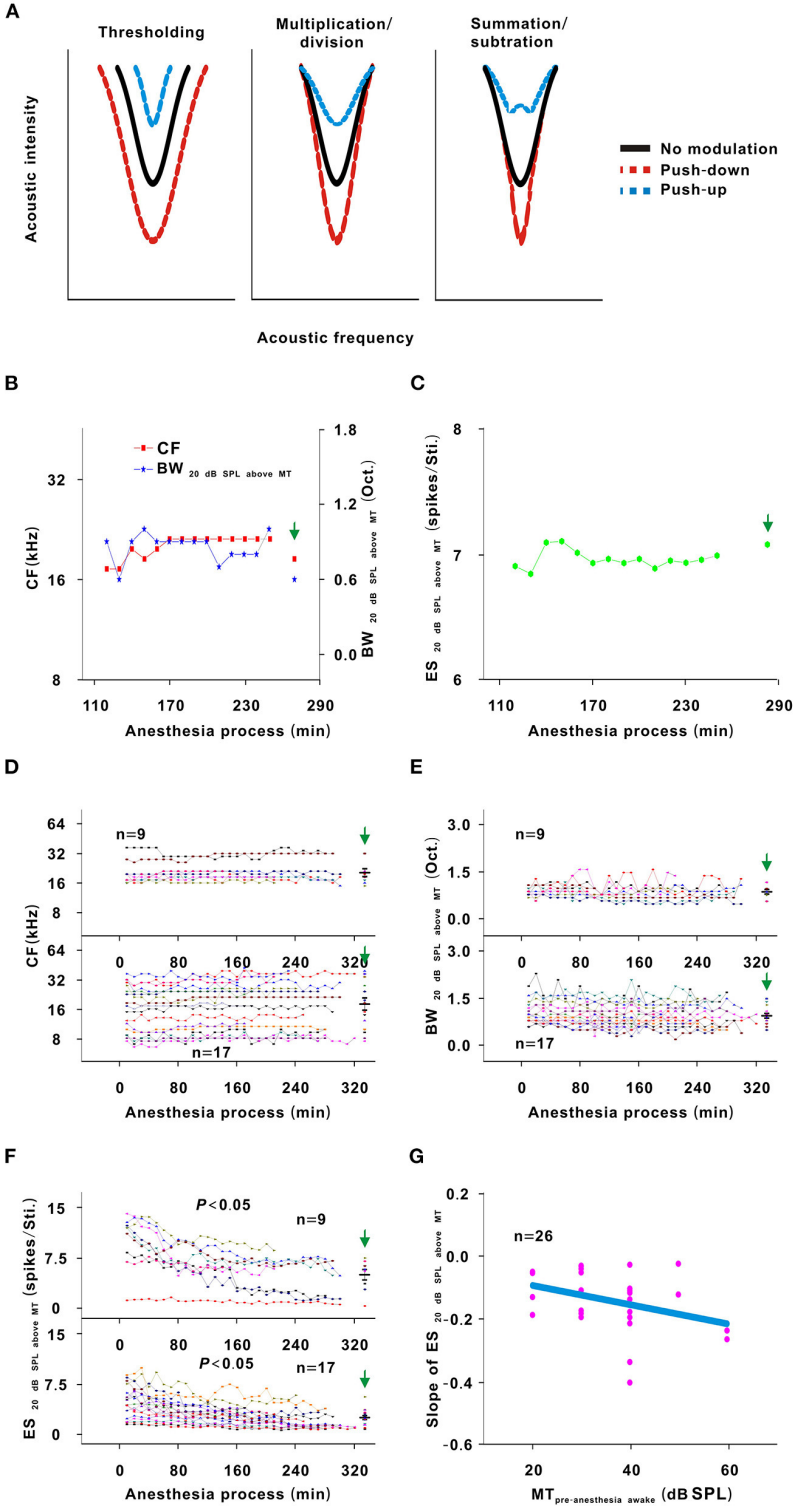
Changes in the TRF of neurons. **(A)** Three potential models for TRF change. Each curve was the contour of the TRF. The left, middle and right sketch maps, respectively, indicated the thresholding, multiplication/division and summation/subtraction models. Black solid line indicated the contour of the TRF with no modulation. Red dotted line indicated the contour of the TRF with push-down. Blue dotted line indicated the contour of the TRF with push-up. **(B)** CF and _above MT_ changes with anesthesia time (red curve and left ordinate for the CF, blue curve and right ordinate for the BW_20 dB SPL aboveMT_); hereafter, the CF- and BW_20 dB SPL above MT_-time curves. **(C)** ES_20 dB SPLabove MT_ changes with anesthesia time; hereafter, the ES_20 dB SPL aboveMT_-time curves. **(D)** Summary of the CF-time curves in neurons_changed MT_ (upper curves) and neurons_unchanged MT_ (lower curves) [Upper curves: first four recording sessions vs. middle four recording sessions vs. last four recording sessions vs. pre-anesthesia awake state (20.957 ± 6.791) vs. (21.682 ± 5.407) vs. (21.415 ± 6.525) vs. (21.072 ± 6.462) kHz, *F* = 0.025, *P* = 0.995, one-way ANOVA; Lower curves: first four recording sessions vs. middle four recording sessions vs. last four recording sessions vs. pre-anesthesia awake state (17.795 ± 9.182) vs. (19.036 ± 9.640) vs. (19.396 ± 10.212) vs. (21.001 ± 11.621) kHz, *F* = 0.285, *P* = 0.836, one-way ANOVA]. **(E)** Summary of the BW_20 dB SPL above MT_-time curves in neurons_changed MT_ (upper curves) and neurons_unchanged MT_ (lower curves) [Upper curves: first four recording sessions vs. middle four recording sessions vs. last four recording sessions vs. pre-anesthesia awake state (0.919 ± 0.130) vs. (0.856 ± 0.221) vs. (0.894 ± 0.181) vs. (0.900 ± 0.224) Oct., *F* = 1.174, *P* = 0.913, one-way ANOVA; Lower curves: first four recording sessions vs. middle four recording sessions vs. last four recording sessions vs. pre-anesthesia awake state (1.037 ± 0.354) vs. (0.954 ± 0.357) vs. (0.912 ± 0.336) vs. (0.953 ± 0.310) Oct., *F* = 0.404, *P* = 0.751, one-way ANOVA]. **(F)** Summary of the ES_20 dB SPL above MT_-time curves in neurons_changed MT_ (upper curves) and neurons_unchanged MT_ (lower curves) [Upper curves: first four recording sessions vs. middle four recording sessions vs. last four recording sessions vs. pre-anesthesia awake state (9.637 ± 3.851) vs. (6.084 ± 2.734) vs. (5.030 ± 2.934) vs. (5.101 ± 2.325) spikes/Sti., *F* = 4.668, *P* = 0.008, one-way ANOVA, Multiple comparisons (LSD): *P* = 0.003 for first four recording sessions vs. last four recording sessions, *P* = 0.003 for first four recording sessions vs. pre-anesthesia awake state; Lower curves: first four recording sessions vs. middle four recording sessions vs. last four recording sessions vs. pre-anesthesia awake state (4.584 ± 2.277) vs. (3.005 ± 1.525) vs. (2.280 ± 1.357) vs. (2.484 ± 1.203) spikes/Sti., *F* = 6.849, *P* = 4.493 × 10^−4^, one-way ANOVA, Multiple comparisons (Tamhane's T2): *P* = 0.008 for first four recording sessions vs. last four recording sessions, *P* = 0.015 for first four recording sessions vs. pre-anesthesia awake state]. **(G)** Scatterplot for the slopes of the ES_20 dB SPL above MT_-time curves against the MT_pre−anesthesia awake_ and the fitting curve to a linear equation (*P* = 0.043, *R*^2^ = 0.081, intercept = −0.031, slope = −0.003).

In the third representative neuron, according to the TRF color, this neuron's response recovered to its strongest at 120 min after anesthesia (Figure 6). From 120 to 250 min, although the MT slowly increased, the contour of the TRF bottom seemed to remain relatively stable (Figure 6, blue and green curves). To demonstrate this point, the TRF parameters (CF, BW_20 dB SPL above MT_, and ES_20 dB SPL above MT_) (Liang et al., 2014) were extracted. In this neuron, the CF and BW_20 dB SPL above MT_ did not obviously change (Figure 7B). The same change regularities were observed in all neurons during anesthesia (*P* > 0.05; Figures 7D,E). For all neurons, there were no significant differences in CF and BW_20 dB SPL above MT_ between in the first four recording sessions and in the pre-anesthesia awake state (*P* > 0.05; Figures 7D,E). This meant that urethane could “push down” the TRF without altering its shape in the thresholding model (Liang et al., 2014) in neurons_changed MT_ (Figures 4, 6) but did not change the TRF in neurons_unchanged MT_ (Figure 2). In addition, in all neurons, the ES_20 dB SPL aboveMT_ presented a decreasing trend during anesthesia (*P* < 0.05; Figures 7C,F). In first four recording sessions, the ES_20 dB SPLabove MT_ was higher than that in the pre-anesthesia awake state (*P* < 0.05; Figure 7F). Similarly, the slope of the ES_20 dB SPL aboveMT_-time curve slightly decreased with the MT_pre−anesthesia awake_ (Figure 7G).

### Effect of Urethane on the FSL and SS

**Figure 8:**
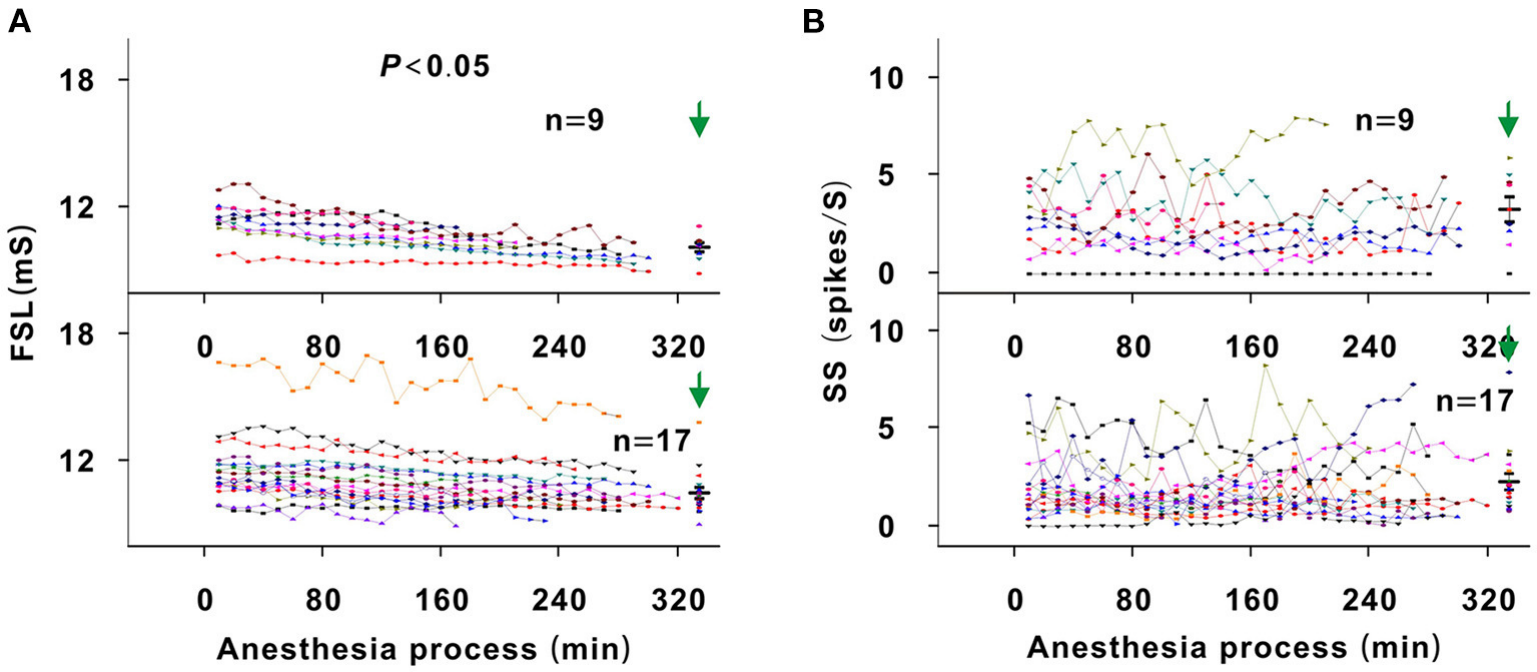
Changes in the FSL and SS of neurons. **(A)** Summary of FSL changes with anesthesia time in neurons_changedMT_ (upper curves) and neurons_unchangedMT_ (lower curves) [Upper curves: first four recording sessions vs. middle four recording sessions vs. last four recording sessions vs. pre-anesthesia awake state (11.454 ± 0.888) vs. (10.674 ± 0.697) vs. (10.158 ± 0.642) vs. (10.123 ± 0.621) ms, *F* = 6.702, *P* = 0.001, one-way ANOVA, Multiple comparisons (LSD): *P* = 0.001 for first four recording sessions vs. last four recording sessions, *P* = 4.356 × 10^−4^ for first four recording sessions vs. pre-anesthesia awake state; Lower curves: first four recording sessions vs. middle four recording sessions vs. last four recording sessions vs. pre-anesthesia awake state (11.622 ± 1.612) vs. (11.044 ± 1.447) vs. (10.755 ± 1.234) vs. (10.497 ± 1.096) ms, *F* = 2.140, *P* = 0.104, one-way ANOVA]. **(B)** Summary of SS changes with anesthesia time in neurons_changedMT_ (upper curves) and neurons_unchangedMT_ (lower curves) [Upper curves: first four recording sessions vs. middle four recording sessions vs. last four recording sessions vs. pre-anesthesia awake state (2.751 ± 1.654) vs. (2.479 ± 1.783) vs. (2.765 ± 2.175) vs. (3.298 ± 1.919) spikes/S, *F* = 0.295, *P* = 0.829, one-way ANOVA; Lower curves: first four recording sessions vs. middle four recording sessions vs. last four recording sessions vs. pre-anesthesia awake state (2.060 ± 1.554) vs. (1.725 ± 1.182) vs. (2.086 ± 1.628) vs. (2.178 ± 1.765) spikes/S, *F* = 0.279, *P* = 0.841, one-way ANOVA].

Theoretically, as urethane is metabolized, the neuronal response to tone should gradually increase (Albrecht and Davidowa, 1989). However, the neuron response decreased in this study. Namely, urethane could improve the response of auditory neurons to tone. To analyze the improvement mechanism, the FSL and SS were investigated. In all neurons, the SS remained relatively steady during anesthesia (*P* > 0.05; Figure 8B). For first four recording sessions vs. pre-anesthesia awake state, the SS values were similar (*P* > 0.05; Figure 8B). The FSL gradually decreased with anesthesia time in neurons_changed MT_ (*P* < 0.05), there was no statistical significance in FSL change in neurons_unchanged MT_ (*P* > 0.05; Figure 8A). In neurons_changed MT_, the FSL increased in the first four recording sessions in comparison with that in the pre-anesthesia awake state (*P* < 0.05; Figure 8A). For first four recording sessions vs. preanesthesia awake state, the FSL of neurons_unchanged MT_ were similar (*P* > 0.05; Figure 8A). Thus, the changes in SS and FSL reflected that the inhibitory effect of urethane was not weakened in the recording session with the strongest neuron response. Therefore, the gradual decreased neuron response (e.g., the neuron in Figure 4) was not due to the direct inhibitory effect of urethane.

### Changes in Neuron Response Induced by the Direct Inhibitory Effect of Urethane

**Figure 9:**
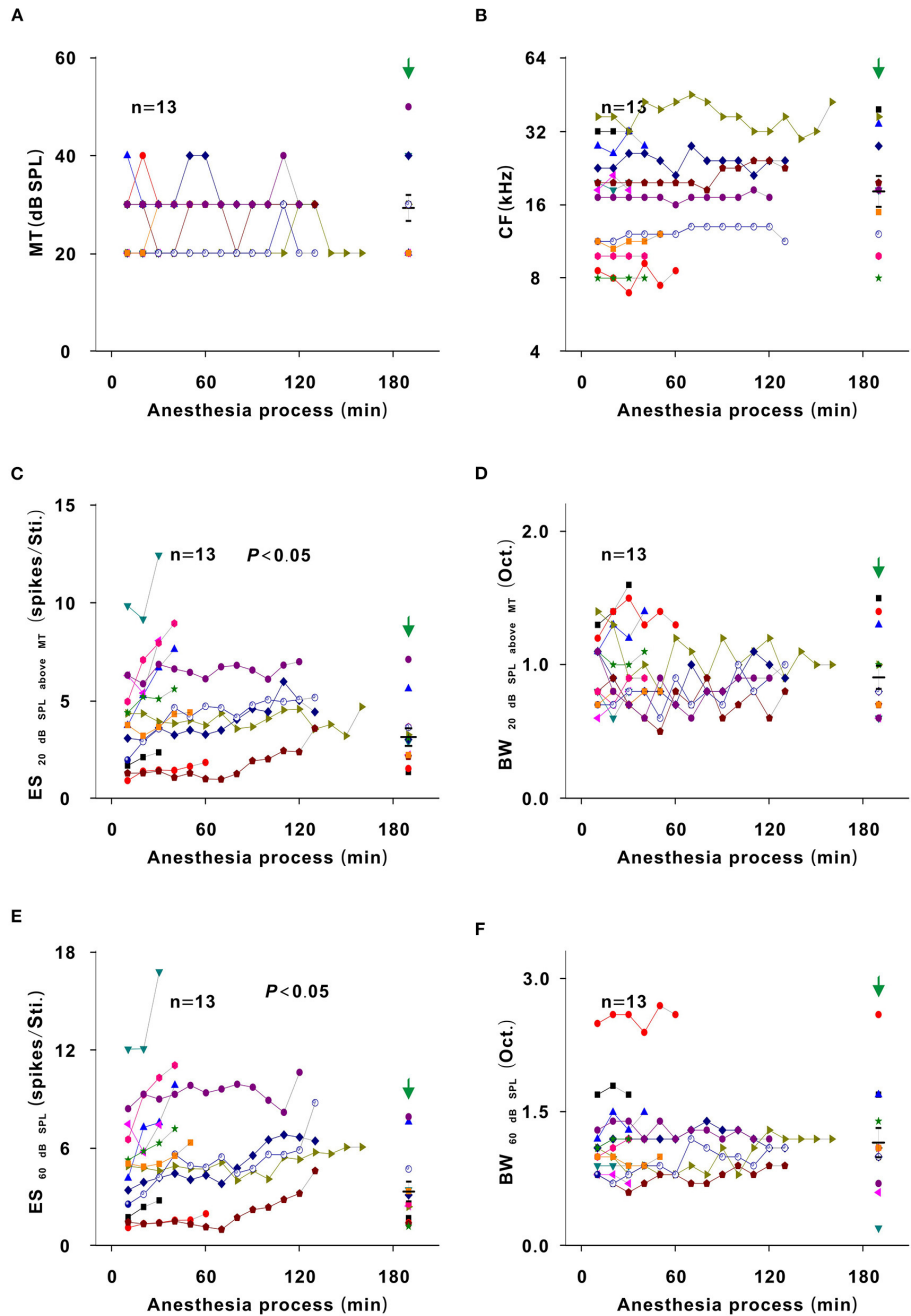
Inhibitory effect of urethane induced changes in the neuron response. Changes in MT **(A)**, CF **(B)**, ES_20dBSPLaboveMT_ **(C)**, BW_20dBSPLaboveMT_ **(D)**, ES_60dBSPL_ **(E)**, and BW_60dBSPL_ **(F)** in the course of the initial gradual increase in neuron response [first recording session vs. recording session with the strongest response, paired *t*-test, MT: (24.615 ± 6.602) vs. (24.615 ± 5.189) dB SPL, *t* = 0.000, *P* = 1.00; CF: (13.687 ± 4.853) vs. (14.104 ± 4.957) kHz, *t* = −1.819, *P* = 0.094; ES_20dBSPLaboveMT_: (4.047 ± 2.477) vs. (5.811 ± 2.929) spikes/Sti., *t* = −4.728, *P* = 4.905 × 10^−4^; BW_20dBSPLaboveMT_: (0.977 ± 0.255) vs. (1.023 ± 0.259) Oct., *t* = −0.776, *P* = 0.453; ES_60dBSPL_: (4.962 ± 3.122) vs. (7.708 ± 3.883) spikes/Sti., *t* = −4.960, *P* = 3.309 × 10^−4^; BW_60dBSPL_: (1.177 ± 0.468) vs. (1.254 ± 0.479) Oct., *t* = −2.132, *P* = 0.054]. The first recording session of each curve was the first recording session after urethane injection (i.e., the recording session at 10 min in each neuron). The last recording session of each curve was the recording session with the strongest response.

In most recording neurons of this study, because there is the improved effect of urethane, the neuron responses in most of recording sessions under the urethane anesthesia state were stronger than those under the pre-anesthesia awake state (Figures 3E, 9C,E). For observing the direct inhibitory effect of urethane, the comparison of neuron response between in the urethane anesthesia state and in the pre-anesthesia awake state was inappropriate. In addition to some neurons that showed a gradual decreasing trend in response to tone (Figure 4), 13 neurons presented an initial increase and then a gradual decrease in response to tone (Figures 2, 6). In the course of the initial gradual increase, we can investigate the direct inhibitory effect of urethane in the 13 neurons. While the ES_60 dB SPL_ and ES_20 dB SPL above MT_ gradually increased to their highest levels (*P* < 0.05), the MT, CF, BW_20 dB SPL above MT_, and BW_60 dB SPL_ did not obviously change (*P* > 0.05; Figure 9). For changes resulting from the inhibitory (Figure 9) and improved (Figure 7) effects, the change regularities were similar, but the change directions were contrary.

### Changes in Neuron Response in an Awake State Without Urethane Anesthesia

**Figure 10:**
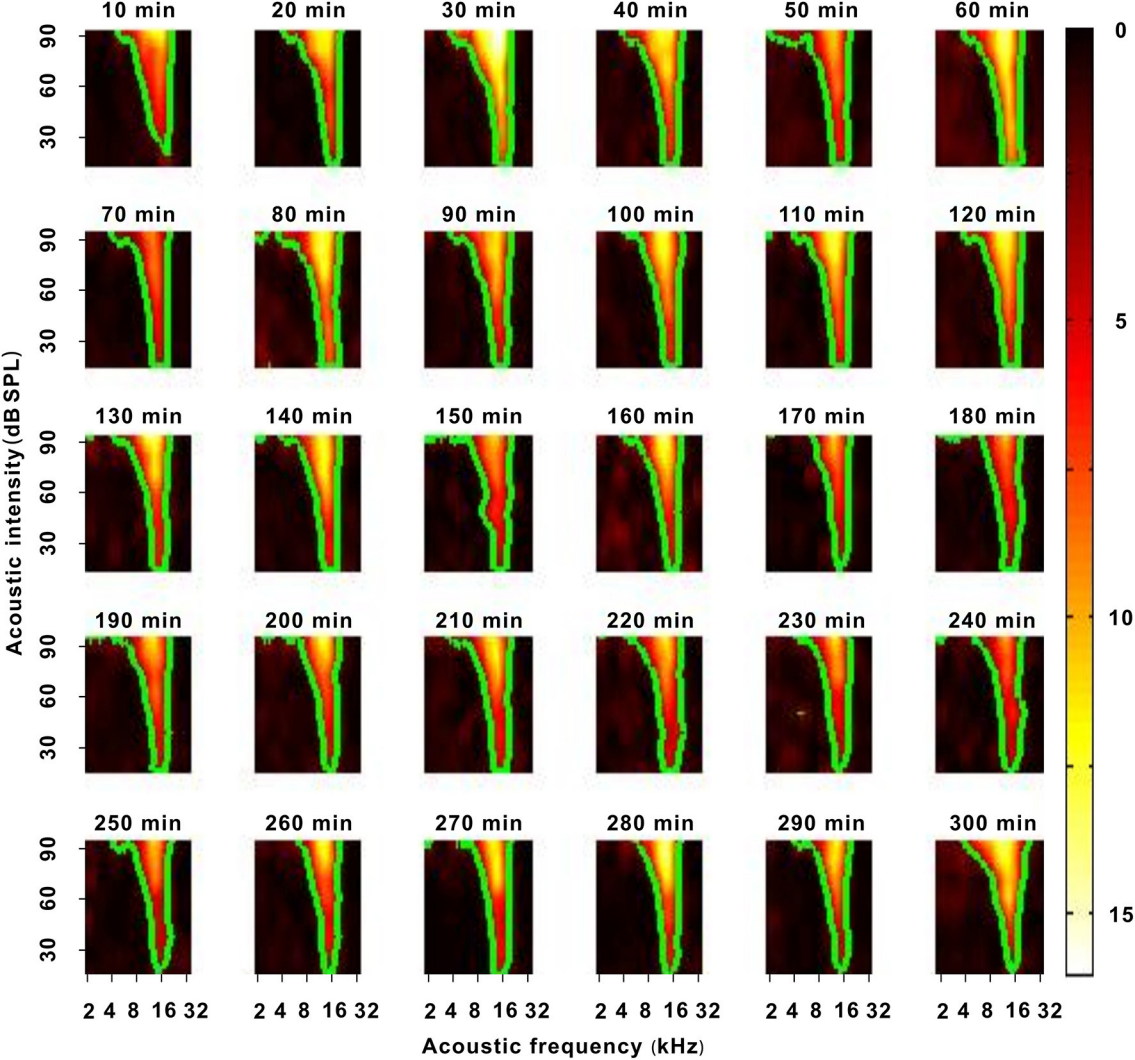
A representative neuron with a change in neuron response in an awake state without urethane anesthesia. Each green curve was the contour of the TRF.

**Figure 11:**
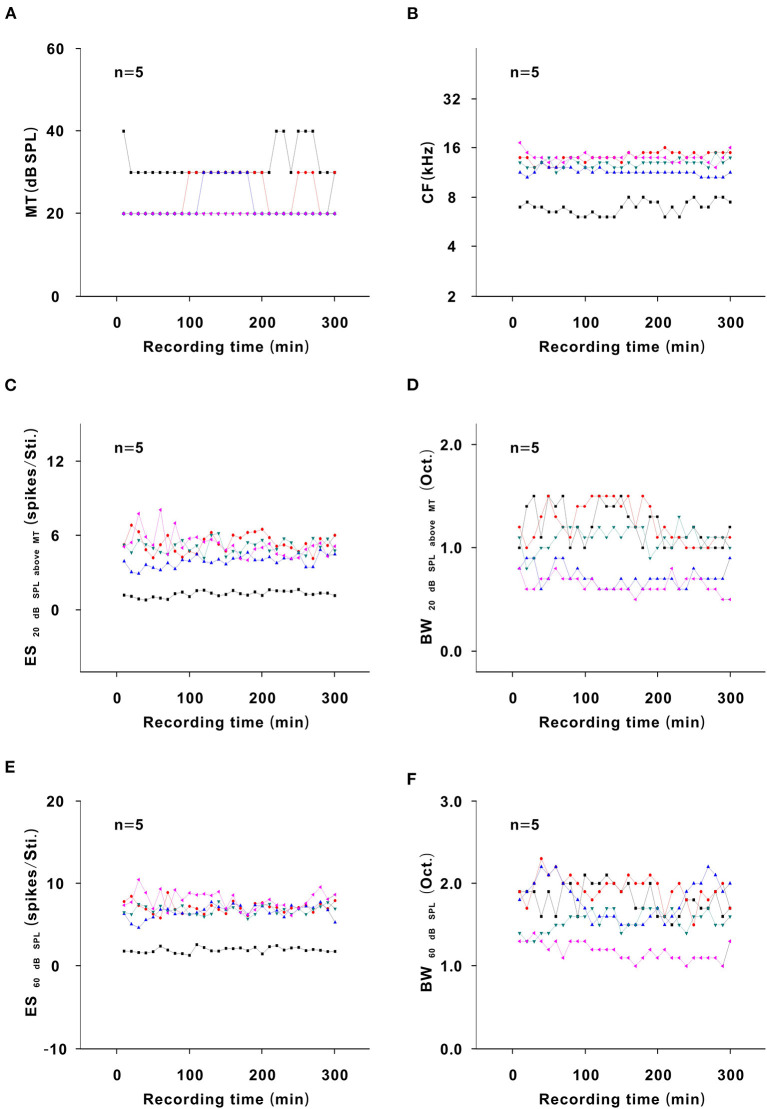
Changes in neuron response in an awake state without urethane anesthesia. **(A)** Summary of the MT-time curves in neurons [first four recording sessions vs. middle four recording sessions vs. last four recording sessions (22.500 ± 5.590) vs. (26.000 ± 5.477) vs. (23.500 ± 5.477) dB SPL, *F* = 0.534, *P* = 0.599, one-way ANOVA]. **(B)** Summary of the CF-time curves in neurons [first four recording sessions vs. middle four recording sessions vs. last four recording sessions (11.860 ± 2.944) vs. (11.743 ± 2.918) vs. (12.081 ± 2.937) kHz, *F* = 0.017, *P* = 0.983, one-way ANOVA]. **(C)** Summary of the ES_20dBSPLaboveMT_-time curves in neurons [first four recording sessions vs. middle four recording sessions vs. last four recording sessions (4.270 ± 2.119) vs. (4.119 ± 1.662) vs. (4.224 ± 1.709) spikes/Sti., *F* = 0.009, *P* = 0.991, one-way ANOVA]. **(D)** Summary of the BW_20dBSPLaboveMT_-time curves in neurons [first four recording sessions vs. middle four recording sessions vs. last four recording sessions (0.956 ± 0.238) vs. (1.030 ± 0.391) vs. (0.900 ± 0.239) Oct., *F* = 0.237, *P* = 0.792, one-way ANOVA]. **(E)** Summary of the ES_60dBSPL_-time curves in neurons [first four recording sessions vs. middle four recording sessions vs. last four recording sessions (6.038 ± 2.699) vs. (6.221 ± 2.353) vs. (6.342 ± 2.626) spikes/Sti., *F* = 0.018, *P* = 0.982, one-way ANOVA]. **(F)** Summary of the BW_60dBSPL_-time curves in neurons [first four recording sessions vs. middle four recording sessions vs. last four recording sessions (1.690 ± 0.328) vs. (1.610 ± 0.357) vs. (1.665 ± 0.348) Oct., *F* = 0.070, *P* = 0.932, one-way ANOVA].

To exclude other potential factors influencing the neuron response, five neurons were recorded for 300 min in the same way in an awake state without urethane anesthesia. In the representative neuron, the color and contour of the TRF did not obviously change with time (Figure 10). For all neurons, the MT, CF, ES_20 dB SPL aboveMT_, BW_20 dB SPL above MT_, ES_60 dB SPL_, and BW_60 dB SPL_ and similarly remained unchanged over time (*P* > 0.05; Figure 11). Namely, there were no other factors that influenced the neuron response to tone.

## Discussion

### Urethane Is Suitable for Investigating Improved Effect of General Anesthetic on Neuronal Function

During the induction and recovery of anesthesia, some patients become more excited (Guedel, 1937; Sachdev and Kruk, 1996). This implies that neuronal function is enhanced. Urethane has an effect on multiple receptor systems (Hara and Harris, 2002). During urethane anesthesia, even small functional changes in multiple receptor systems are enough to induce anesthesia (Koblin, 2002). Therefore, it is believed that urethane hardly inhibits electrophysiological activities (Maggi and Meli, 1986). In addition, a single injection of urethane can provide several hours of anesthesia (Koblin, 2002). Therefore, urethane is suitable to investigate the improved effect of general anesthetic on neuronal function.

### Urethane Improves the Response of Auditory Neurons to Tone

In an awake state without urethane anesthesia, there was no obvious change in the neuronal response to tone over time (Figures 10, 11). Under urethane anesthesia, the MT was reduced, the ES was increased, and the frequency tuning range was broadened by urethane (Figures 3C,E, 5D). In comparison with those in the pre-anesthesia awake state, the MT was lower, the ES was higher, and the frequency tuning range was broader in the urethane anesthesia state (Figures 3C,E, 5D). This means that the response of auditory neurons to tone was improved by urethane, which supports the excitatory phenomenon during the induction and recovery of anesthesia (Xu et al., 2017). Three mechanisms may be used to interpret the improved effect of urethane. First, the excitability of neurons increases. Second, the total external input from dendrites to neurons increases. Third, although the total external input to neurons decreases, the ratio of excitatory/inhibitory postsynaptic inputs increases. The excitability of a neuron can be reflected by its SS. In our previous study, the FSL decreased as the acoustic intensity increased (Tan et al., 2008). That is, the FSL can reflect the intensity of external input to neurons. In the recording session with the strongest neuron response, neither the excitability of the neuron nor the external input to the neuron increased (Figures 8A,B), and the inhibitory effect of urethane did not weaken. Therefore, the improved effect of urethane was likely due to the increase in the ratio of excitatory/inhibitory postsynaptic input. This may be related to urethane inducing abnormalities in the cortico-subcortical neuronal circuits (Sachdev and Kruk, 1996).

In this study, the response in neurons with low MT_pre−anesthesia awake_ values was more difficult to be changed (Figures 3D,F, 5E). Lower MT_pre−anesthesia awake_ values mean that the neurons were more excitatory. The improved effect of urethane may be relatively smaller and induce less neuron response. The inhibitory effect of urethane decreases the SS (Albrecht and Davidowa, 1989; Capsius and Leppelsack, 1996), which is different from the finding that the improved effect of urethane did not change the SS (Figure 8B). The SS is usually low in ICC neurons. The improved effect of urethane may also be relatively small and was not enough to cause SS changes.

### Urethane “Pushes Down” the TRF of Auditory Neurons in the Thresholding Model

For neurons_unchanged MT_, the BW_60 dB SPL_, BW_20 dB SPL above MT_, and CF remained stable under urethane anesthesia (Figures 5D, 7D,E), and the BW_60 dB SPL_, BW_20 dB SPL above MT_, and CF were similar when comparing the urethane anesthesia state with the pre-anesthesia awake state (Figures 5D, 7D,E). In these neurons, urethane did not change the TRF. For neurons_changed MT_, the BW_60 dB SPL_ was broadened by urethane, and the BW_20 dB SPL above MT_ and CF remained unchanged under urethane anesthesia (Figures 5D, 7D,E). Similar regularities in the pattern of changes were observed when comparing the urethane anesthesia state with the pre-anesthesia awake state (Figures 5D, 7D,E). Namely, when the MT of neurons was lowered by urethane (Figure 3C), the frequency tuning bandwidth at a fixed acoustic intensity (BW_60 dB SPL_) was broadened (Figure 5D), and the contour of the TRF was pushed down without a change in the shape of the bottom, which was reflected by BW_20 dB SPL above MT_ and CF changes (Figures 7D,E).

Therefore, the TRF of neurons was “pushed down” by the improved effect of urethane in the thresholding model (Xiong et al., 2013). This is similar to the TRF change induced by noise (Liang et al., 2014). In auditory midbrain, the inhibitory effect of urethane did not influence the frequency tuning bandwidth at a fixed acoustic intensity (90, 70, or 20 dB SPL) (Schumacher et al., 2011). The TRF seemed to be altered in other models. The different change models may be due to species differences. Whether the CF or BF were not changed by the improved effect of urethane (Figures 5C, 7D), which was similar to the inhibitory effect of urethane (Schumacher et al., 2011) and other general anesthetics (Gaese and Ostwald, 2001). This is because the CF and BF of neurons mostly depend on neuron location in the auditory system (Kiang and Moxon, 1972; Casseday and Covey, 1992).

### Inhibitory Effect of Urethane on Neurons

In some neurons, the response of auditory neurons to tone was first improved and then gradually decreased (Figures 2, 6). However, the FSL consistently and gradually decreased (Figure 8A). The initial change in the response of auditory neurons from weak to strong should be due to the gradual decrease in the inhibitory effect of urethane. As a general anesthetic, it is reasonable that urethane has inhibitory effect on neurons (Hara and Harris, 2002). In previous studies, in cochlea (Fu et al., 2016), auditory midbrain (Schumacher et al., 2011), dorsal lateral geniculate body (Albrecht and Davidowa, 1989) or auditory cortex (Capsius and Leppelsack, 1996; Gaese and Ostwald, 2001), urethane shows a direct inhibitory effect on response intensity of outer hair cells or neurons, but urethane does not influence CF and/or BF. This supports the direct inhibitory effect of urethane on neurons in inferior colliculus in this study (Figure 9C). In auditory midbrain, the frequency tuning bandwidth at a fixed acoustic intensity (90, 70, or 20 dB SPL) was not influenced by the inhibitory effect of urethane (Schumacher et al., 2011). This is inconsistent with the direct inhibitory effect of urethane on neurons in inferior colliculus in this study (Figure 9), and may be also attributed to species differences.

In addition, in previous studies, the inhibitory effect of urethane was usually observed. This may be partly attributed to that the data is collected during the change in the response of auditory neurons from weak to strong, or is collected from the different recording neurons. The change regularity caused by the inhibitory effect was similar to that caused by the improved effect, but the change directions caused by both effects were opposite (Figures 7, 9). Urethane can decrease the ratio of excitatory/inhibitory postsynaptic inputs (Hara and Harris, 2002) and induce a direct inhibitory effect. The improved effect of urethane might result from an increase in the ratio of excitatory/inhibitory postsynaptic inputs. Therefore, it was reasonable that the inhibitory and improved effects were similar.

### Strengths and Limitations

Evidence suggests that general anesthetics can improve the response of neurons (Guedel, 1937; Huang et al., 2015; Xu et al., 2017). However, it is unclear how general anesthetics improve the response of neurons. The strength of this study is that we elucidated the characteristics of the improved effect of urethane on the response of neurons.

This study has some limitations. First, the mechanism of action of urethane is different from that of other general anesthetics (Patel and Goa, 1996; Hara and Harris, 2002; Sahinovic et al., 2018). It is unclear how other common general anesthetics (e.g., propofol and sevoflurane) change the response of neurons. Second, this study was carried out in mice. The information processing mechanism of neurons in mice may be different from that in humans (Waguespack et al., 2020). Third, this study was carried out on the auditory system. There are different information processing mechanisms of neurons among different sensory systems (Zhou et al., 2010; Liu et al., 2011). Fourth, excitatory (or inhibitory) postsynaptic inputs to neurons can be obtained in a whole-cell recording manner (Wehr and Zador, 2003). However, long-term whole-cell recording is very difficult (Yan et al., 2020). We could not provide direct evidence that urethane increases the ratio of excitatory/inhibitory postsynaptic inputs.

## Conclusions

Urethane improves the response of auditory neurons to tone by lowering/not changing the MT, enhancing the ES, or increasing/not changing the frequency tuning range. Urethane “pushes down” the TRF in the thresholding model or does not change the TRF. The improved effect increases as the MT_pre−anesthesia awake_ of neurons increases. The change regularities resulting from the inhibitory and improved effects of urethane are similar, but the change directions are contrary. An increase in the ratio of excitatory/inhibitory postsynaptic inputs to neurons may be the reason for the improvement mechanism.

## Data Availability Statement

The original contributions presented in the study are included in the article/supplementary material, further inquiries can be directed to the corresponding authors.

## Ethics Statement

The animal study was reviewed and approved by the Animal Care and Use Committee of Southern Medical University.

## Author Contributions

ZX and JH conceived and designed the experiments. BH, LY, and YL performed the experiments. BH, WL, and ML analyzed the data. ZX contributed reagents, materials, and analysis tools. JH revised the paper. All authors contributed to the article and approved the submitted version.

## Funding

This work was supported by grants from the National Natural Science Foundation of China to ZX (31872769 and 32070994).

## Conflict of Interest

The authors declare that the research was conducted in the absence of any commercial or financial relationships that could be construed as a potential conflict of interest.

## Publisher's Note

All claims expressed in this article are solely those of the authors and do not necessarily represent those of their affiliated organizations, or those of the publisher, the editors and the reviewers. Any product that may be evaluated in this article, or claim that may be made by its manufacturer, is not guaranteed or endorsed by the publisher.
